# Supplementary material for: AIRR-C IG Reference Sets: curated sets of immunoglobulin heavy and light chain germline genes
Source: Front Immunol. 2024 Feb 9;14:1330153. doi: 10.3389/fimmu.2023.1330153 (PMC10884231; doi:10.3389/fimmu.2023.1330153)
Supplement: Supplementary Table 1 — Evidence in support of the existence of human IGH genes that were candidates for inclusion in the AIRR-C IGH_VJ Reference Set, but which lacked sufficient evidence for inclusion. [file Table_1.pdf]

Supplementary Table I: Evidence in support of the existence of human IGH genes that were candidates for inclusion in the AIRR-C IGH_VJ Reference Set, but which lacked sufficient evidence for inclusion.

**Gene Name**	**Matsuda[1]**	**Scheepers[2]**	**Wang[3]**	**Rodriguez[4]**	**Gidoni-VDJbase[5]**
IGHV1-45*01	M99645				
IGHV1-69*16		MG719328			
IGHV2-5*05				Y	
IGHV2-26*03		MG719336			
IGHV2-26*05				Y	
IGHV2-70*03					
IGHV2-70*13	AB019437				
IGHV2-70*16		MG719341			
IGHV2-70*17		MG719342			
IGHV3-13*02	M99653				
IGHV3-15*02	M99654				
IGHV3-16*01	M99655				
IGHV3-21*04			HM855720		
IGHV3-25*01	M99661				
IGHV3-33*02	M99665				
IGHV3-47*03	M99674				
IGHV3-49*01	M99676				
IGHV3-62*01	AB019437				
IGHV4-28*06			HM855925		
IGHV4-30-2*03					P1_I55[6]
IGHV4-31*02	M99683				
IGHV4-34*02	M99684				
IGHV4-59*13				AC279155	
IGHV4-61*08	AB019437				

[1] GenBank accession numbers of sequences from the studies of Matsuda and colleagues

[2] GenBank accession numbers of sequences from the studies of Scheepers and colleagues

[3] GenBank accession numbers of sequences from the studies of Wang and colleagues

[4] GenBank accession numbers of sequences from the studies of Rodriguez and colleagues.
'Y' indicates observed in the study, but not reported in GenBank.
[5] Dataset from the study of Gidoni and colleagues, available from the VDJbase website
[6] Observed as a single exact match in AIRR-seq data
