# Supplementary material for: AIRR-C IG Reference Sets: curated sets of immunoglobulin heavy and light chain germline genes
Source: Front Immunol. 2024 Feb 9;14:1330153. doi: 10.3389/fimmu.2023.1330153 (PMC10884231; doi:10.3389/fimmu.2023.1330153)
Supplement: Supplementary Table 2 — Evidence in support of the existence of human IGKV genes that were candidates for inclusion in the AIRR-C IGKappa_VJ Reference Set, but which lacked sufficient evidence for inclusion. [file Table_2.pdf]

Supplementary Table II: Evidence in support of the existence of human IGKV genes that were candidates for inclusion in the AIRR-C IGKappa_VJ Reference Set, but which lacked sufficient evidence for inclusion.

	Zachau[1]	Winter[2]	Watson[3]	Kawasaki[4]	Gidoni[5]
IGKV1-6*02			GRCh38.p14		
IGKV1D-12*02			AC243981		
IGKV1D-13*01				NG_000833	
IGKV1D-8*03					P1_I74
IGKV2-4*01	X72814				
IGKV2D-26*02		Z27499			
IGKV3-7*02	X72812				

[1] GenBank accession numbers of sequences from the studies of Zachau and colleagues
[2] GenBank accession numbers of sequences from the studies of Winter and colleagues
[3] GenBank accession numbers of sequences from the studies of Watson and colleagues
[4] GenBank accession numbers of sequences from the studies of Kawasaki and colleagues
[5] Datasets from the study of Gidoni and colleagues, available from the VDJbase website
