# Supplementary material for: AIRR-C IG Reference Sets: curated sets of immunoglobulin heavy and light chain germline genes
Source: Front Immunol. 2024 Feb 9;14:1330153. doi: 10.3389/fimmu.2023.1330153 (PMC10884231; doi:10.3389/fimmu.2023.1330153)
Supplement: Supplementary Table 3 — Evidence in support of the existence of human IGLV genes that were candidates for inclusion in the AIRR-C IGLambda_VJ Reference Set, but which lacked sufficient evidence for inclusion. [file Table_3.pdf]

Supplementary Table III: Evidence in support of the existence of human IGLV genes that were candidates for inclusion in the AIRR-C IGLambda_VJ Reference Set, but which lacked sufficient evidence for inclusion.

	VDJbase Genomic[1]	Winter et al[2]	Kawasaki et al[3]
IGLV1-40*02		Z22193	
IGLV1-40*03		Z22192	
IGLV1-44*04	P28_I6		
IGLV1-47*03	P28_I5		
IGLV10-54*06	P28_I5		
IGLV2-11*02		Z22198	
IGLV2-8*04	P28_I3		
IGLV3-1*02	P28_I3		
IGLV3-22*02			
IGLV3-25*01		X97474	
IGLV4-60*01		Z73667	
IGLV5-45*05	P28_I6		
IGLV7-46*05	P28_I6		
IGLV9-49*02			D87016

[1] Datasets from the study of Gibson and colleagues, available from the VDJbase website

[2]GenBank accession numbers of sequences from the studies of Winter and colleagues

[3] GenBank accession numbers of sequences from the studies of Kawasaki and colleagues
