# Supplementary material for: AIRR-C IG Reference Sets: curated sets of immunoglobulin heavy and light chain germline genes
Source: Front Immunol. 2024 Feb 9;14:1330153. doi: 10.3389/fimmu.2023.1330153 (PMC10884231; doi:10.3389/fimmu.2023.1330153)
Supplement: Supplementary file 4 [file DataSheet_1.docx]

**AIRR-C IG Reference Sets: curated sets of immunoglobulin heavy and light chain germline genes**

Andrew M. Collins, Mats Ohlin, Martin Corcoran, James M. Heather, Duncan Ralph, Mansun Law, Jesus Martínez-Barnetche, Jian Ye, Eve Richardson, William S. Gibson, Oscar L. Rodriguez, Ayelet Peres, Gur Yaari, The AIRR-Community, Corey T. Watson, William D. Lees

This manuscript has been endorsed by the AIRR Community. AC, MO, MC, JH, DR, ML, JM-B, JY, ER, WG, OR, AP, GY, CW and WL are Participants of the AIRR Community Germline Gene Working Group. The following individuals asked to be named as endorsing this manuscript:

| **Name** | **Affiliation** |
| --- | --- |
| Aayush | Universiti Putra Malaysia, Serdang, Malaysia |
| Brian Corrie | Department of Biological Sciences, Simon Fraser University, Burnaby, Canada |
| Brien Haun | The University of Hawaii, Honolulu, USA |
| Chaim Schramm | Vaccine Research Center, National Institute of Allergy and Infectious Diseases, National Institutes of Health, Rockville, USA |
| Christian Busse | Division of B Cell Biology, German Cancer Research Center, Heidelberg, Germany |
| Daniel Douek | Human Immunology Section, National Institutes of Health, Bethesda, USA |
| Dylan Duchen | Yale University School of Medicine, New Haven, USA |
| Edel Aron | Yale University School of Medicine, New Haven, USA |
| Encarnita Mariotti | Sorbonne Universite, France |
| Gregory Ippolito | The University of Texas at Austin, USA |
| Jesse Connell | University of Pennsylvania, Philadelphia, USA |
| Matthias Bruhn | Institute for Experimental Infection Research, TWINCORE, Centre for Experimental and Clinical Infection Research, a joint venture between the Helmholtz Centre for Infection Research and the Hannover Medical School, Hannover, Germany |
| Noah Yann Lee | Yale University School of Medicine, New Haven, USA |
| Parham Habibzadeh | University of Maryland School of Medicine, Baltimore, USA |
| Quy Khang Le | Department of Immunology, University of Oslo, Oslo, Norway |
| Rodrigo García Valiente | Amsterdam UMC, Amsterdam, Netherlands |
| Sakshi Bansal | Postgraduate Institute of Medical Education and Research, Chandigarh, India |
| Sandeep Sehrawat | National Institute of Pharmaceutical Education and Research, SAS Nagar, India |
| Uddalok Jana | University of Louisville, Louisville, USA |
| William Gibson | National Institute of Allergy and Infectious Diseases, National Institutes of Health, Rockville, USA |
| Wim Maes | PharmAbs, the KU Leuven Antibody Center, Leuven, Belgium |
